# Effect of intra‐abdominal hypertension on the intraocular pressure of the conscious dogs

**DOI:** 10.1002/vms3.441

**Published:** 2021-02-01

**Authors:** Min Jang, Won‐Gyun Son, Hyunseok Kim, Chi Won Shin, Inhyung Lee

**Affiliations:** ^1^ Department of Veterinary Clinical Sciences College of Veterinary Medicine Seoul National University Seoul Korea; ^2^ Department of Veterinary Surgery College of Veterinary Medicine Kyungpook National University Daegu Republic of Korea

**Keywords:** dog, intra‐abdominal hypertension, intra‐abdominal pressure, intraocular pressure

## Abstract

This study was performed to evaluate the effect of intra‐abdominal pressure (IAP) on intraocular pressure (IOP) in conscious dog models using a balloon technique to generate intra‐abdominal hypertension. Six healthy dogs without ocular abnormalities were evaluated in this study. A balloon device was placed in the intra‐abdominal cavity. The abdomen was insufflated to IAP levels of 15 and 25 mmHg using the balloon device. Intraocular pressure was measured at baseline, at IAP levels of 15 and 25 mmHg, and after decompression. In comparison with the mean baseline IOP (15.1 ± 2.0 mmHg), there was a significant increase in IOP at IAP levels of 15 mmHg (20.0 ± 2.1 mmHg) and 25 mmHg (19.9 ± 2.2 mmHg), corresponding to a 32.4% and 31.7% increase from baseline IOP, respectively. The mean IOP after decompression (14.8 ± 1.7 mmHg) was significantly lower compared to those at IAP levels of 15 and 25 mmHg. The present findings demonstrate that increased IAP has a clinically significant effect on IOP in dogs under conscious conditions. Although more research is needed to determine of increased IAP on IOP, these findings suggest that increased IAP leads to mild and reversible increase in IOP.

## Graphical abstract

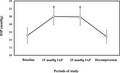

## 1 INTRODUCTION

An acute rise of intra‐abdominal pressure (IAP) leads to intra‐abdominal hypertension (IAH), which if severe and left untreated can be life threatening (Hoareau & Mellema, 2015; Nielsen & Whelan, 2012; Parsak et al., 2008). IAH is defined as a constant elevation of IAP of more than 12 mmHg (Hunter & Damani, 2004). IAH is clinically important because it might lead to impairment of haemodynamic stability, organ perfusion and pulmonary function (Hunter & Damani, 2004; Smith & Sande, 2012; Way & Monnet, 2014). Elevated IAP has been reported to result in increased intrathoracic, intracranial and intraocular pressure (Bloomfield et al., 1997; Deeren et al., 2005; Ece et al., 2015; Halverson et al., 1998).

Intraocular pressure (IOP), defined as pressure exerted by components of the eye against the fibrous tunic, is regulated by the central nervous system, which maintains a balance between aqueous humor production and outflow (Almeida et al., 2008; Kovalcuka et al., 2013). It can be affected by external pressure, scleral rigidity, intraocular changes, arterial blood pressure, central venous pressure, drugs with sympathetic or parasympathetic effect on muscle tone and changes in aqueous humor production and outflow rates (Almeida et al., 2004). Sudden increase in IOP might cause progressive optic nerve damage and severe complications in patients with near‐perforating corneal lesions or glaucoma (Hofmeister et al., 2009; Maślanka, 2015).

Increase in IAP induced by standard pressure pneumoperitoneum (≤14 mmHg) may lead to a mild and reversible increase in IOP (Grosso et al., 2013). Several studies have evaluated the effects of pneumoperitoneum during laparoscopic surgery on IOP and found that pneumoperitoneum causes a significant increase in IOP by up to 10 mmHg and that increase in IOP beyond 10 mmHg can result in optic nerve damage and progressive visual loss (Grosso et al., 2013; Lentschener et al., 1996; Mowafi et al., 2003). However, because these studies were performed in human patients undergoing laparoscopy under general anaesthesia, it is not clear whether the increase in IOP was caused by the effects of pneumoperitoneum, general anaesthesia or posture variation (Bloomfield et al., 1997; Grosso et al., 2013; Halverson et al., 1998; Lentschener et al., 1996). To date, there is no available data regarding the effect of IAH on IOP in conscious dogs.

A previous study has demonstrated the successful increase and maintenance of IAP by air insufflation using a balloon device without general anaesthesia in a conscious IAH dog model (Jang et al., 2018, 2019). This conscious IAH dog model may be helpful for predicting changes in IOP in conscious patients because anaesthetic agents, laparoscopic surgery and posture variation can affect IOP (Grosso et al., 2013; Lentschener et al., 1996; Mowafi et al., 2003).

Therefore, this study aimed to evaluate the effect of acute increases in IAP on IOP in conscious dogs without other interventions.

## 2 MATERIALS AND METHODS

### 2.1 Animals

Six clinically healthy male Beagles (mean age, 2.0 ± 1.0 years) were included after approval by the Institutional Animal Care and Use Committee of Seoul National University (SNU‐160726–6–1). The mean body weight of the included dogs was 9.8 ± 1.3 kg, and the median body condition score on a 9‐point scale was 5 (range, 5–6). Prior to the start of this study, all animals were determined to be free of diseases by thorough physical examination, complete blood count, blood chemistry analysis, thoracic and abdominal radiographic examination. A complete ophthalmologic examination was performed by an experienced ophthalmologist including slit‐lamp biomicroscopy (SL‐D7, Topcon) and indirect ophthalmoscopy (Vantage plus, Keeler).

### 2.2 Preparation of the IAH dog model

Food was withheld for 8 hr before anaesthesia, but water was made available ad libitum. An intravenous catheter was placed in the cephalic vein, and the dog was premedicated with 10 µg/kg intravenous (IV) medetomidine and 4 mg/kg intramuscular tramadol. Anaesthesia was induced with 2 mg/kg IV alfaxalone and maintained with 2% isoflurane in 2 L/min oxygen delivered through a semiclosed circle system. The abdominal region was aseptically prepared for laparotomy. An aseptic latex balloon device for induction of IAH was placed into the abdominal cavity through a 2‐cm ventral midline incision, and the abdominal wall musculature was closed with 2–0 polydioxanone sutures (Jang et al., 2018). Leaving only the urinary catheter port of the device accessible from outside the abdominal cavity, the subcutis and skin were closed with 3–0 polydioxanone and nylon sutures, respectively. A Chinese finger trap suture was used to fix the urinary catheter of the device at the abdominal wall, and the animals were allowed to recover from anaesthesia. The dogs were then administered subcutaneous 8 mg/kg cefovecin sodium and 4 mg/kg carprofen. Postoperative analgesia included the administration of carprofen and tramadol. Rescue analgesia was provided for any dog that was determined to have mild to moderate pain. Intravenous 2 mg/kg tramadol administered to any dog that exhibited a mild to moderate pain and observed for 48 hr for any complications.

### 2.3 Measurement of IOP

Intraocular pressure was measured using a rebound tonometer (TonoVet, Icare). All tonometric measurements were acquired at the axial cornea by the same investigator, with the contact point of the probe being held perpendicularly approximately 4 mm from the cornea. For each dog, IOP of both eyes was measured in the standing position to avoid pressure on the jugular veins and the globe. The average of five IOP measurements, with standard deviation < 5%, was used for further analysis.

### 2.4 Measurement of IAP

Transvesical IAP was measured using the Malbrain technique, with the animals in standing position (Jang et al., 2018; Malbrain, 2004; Smith & Sande, 2012). A Foley urinary catheter was placed in the bladder. Three three‐way stopcocks—the first attached to 1 L of 0.9% sodium chloride solution, the second to a 50‐mL syringe, and the third to a pressure transducer—were connected to the catheter. The bladder was emptied and instilled with 1.0 ml/kg of 0.9% sodium chloride (Way & Monnet, 2014). The pressure transducer was zeroed at the level of the symphysis pubis, following which, IAP measurements were acquired.

### 2.5 Experimental procedures

An interval of 2 days was allowed between installation of the balloon device and IAH induction in order to avoid the residual effects of anaesthetic drugs. Food, but not water, was withheld for 10 hr prior to treatment. Baseline IOP was measured prior to IAH induction, with the dogs in standing position. The abdomen was insufflated to an IAP level of 15 mmHg using the balloon device. The target IAP remained constant after a stabilization period of 60 min and the IOP measurements were repeated. The IAP was then increased to 25 mmHg by additional air insufflation using the balloon device, and the target IAP level was sustained over the next 60 min and the IOP measurements were repeated. Thereafter, the balloon device was deflated, and post‐decompression IOP was measured after a stabilization period of 60 min.

### 2.6 Statistical analysis

Statistical analyses were performed using Statistical Package for the Social Sciences version 22 software for Windows. Changes in IOP over time were evaluated by one‐way analysis of variance with repeated measures. Comparison of IOP of each group at each time point was performed by Bonferroni's *post‐hoc* method. A *p*‐value < .05 was considered statistically significant.

## 3 RESULTS

The IOP values (mean ± *SD*) of the right and left eyes of each dog were measured at each time point (Table 1). There were no significant differences in IOP of the right and left eyes among the different treatments (baseline, at IAP levels of 15 and 25 mmHg, and after decompression). The mean IOP of the right and left eyes was obtained for each dog.

**TABLE 1:** Effect of increased intra‐abdominal pressure (IAP) levels of 15 and 25 mmHg on intraocular pressure (IOP) of both eyes in conscious dogs

Treatment	IOP (mmHg)	
	Left	Right
Baseline	14.7 ± 1.9	15.5 ± 2.3
15 mmHg IAP	19.0 ± 2.0*	21.0 ± 1.9*
25 mmHg IAP	19.5 ± 2.1*	20.3 ± 2.3*
Decompression	14.5 ± 1.9	15.2 ± 1.7

* Statistically significant differences in comparison with baseline values.

**FIGURE 1:**
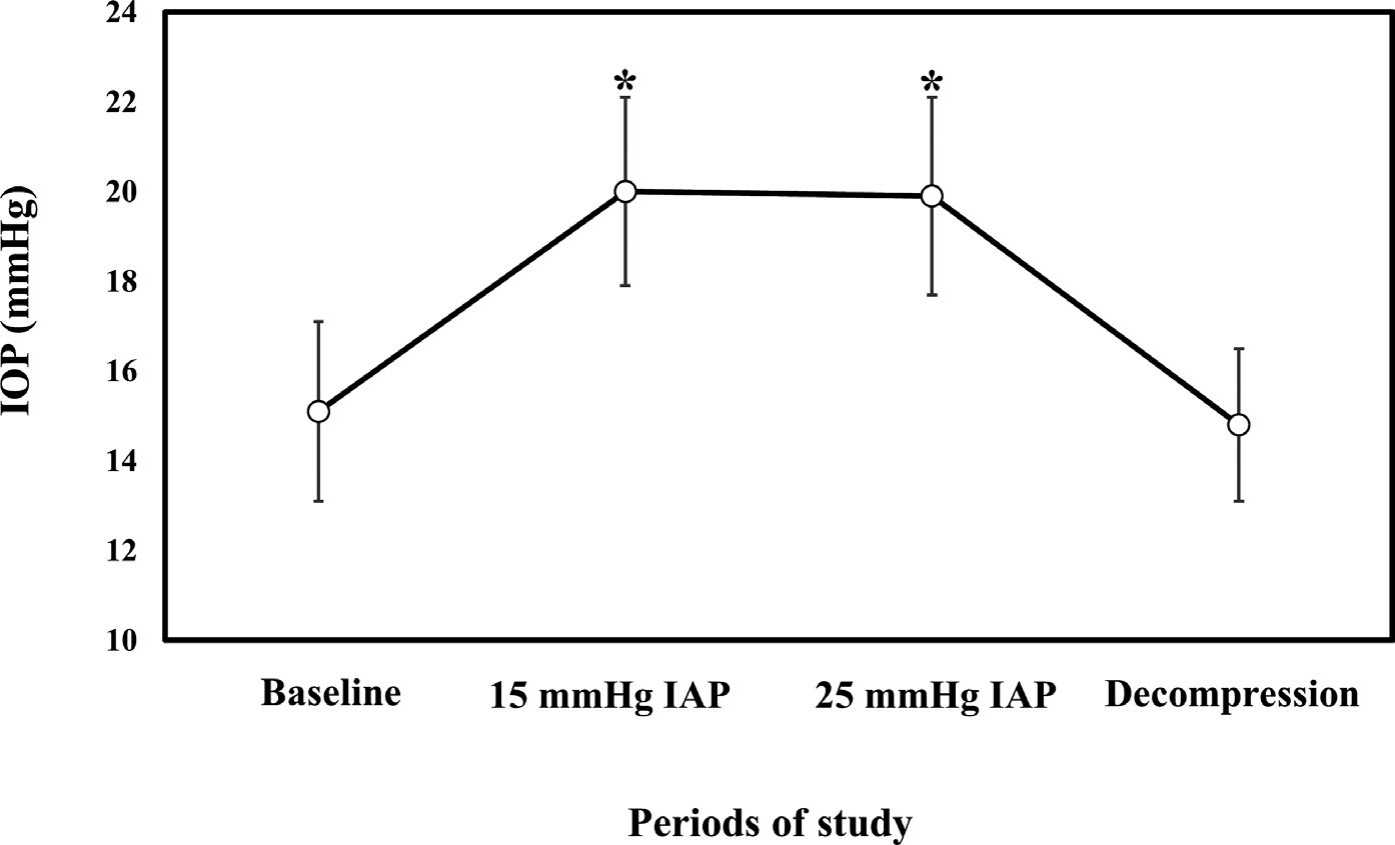
Effect of increased intra‐abdominal pressure (IAP) levels of 15 and 25 mmHg on mean of right and left intraocular pressure (IOP) in conscious dogs. ^*^Statistically significant differences in comparison with baseline values

The mean IAP at baseline was 5.3 ± 1.2 mmHg. In comparison with the mean baseline IOP (15.1 ± 2.0 mmHg), there was a significant increase in IOP at IAP levels of 15 mmHg (20.0 ± 2.1 mmHg; *p* = .00) and 25 mmHg (19.9 ± 2.2 mmHg; *p* = .00), corresponding to a 32.4% and 31.7% increase from baseline IOP, respectively (Figure 1). There was no significant difference in mean IOP at IAP levels of 15 and 25 mmHg. The mean IOP after decompression (14.8 ± 1.7 mmHg) was significantly lower compared to those at IAP levels of 15 and 25 mmHg. No other side‐effects were observed after removal of the balloon device and during a follow‐up period of 2 months.

## 4 DISCUSSION

In this study, increase in IAP to 15 and 25 mmHg caused a significant increase in IOP from the baseline value. These results are consistent with the findings of human studies demonstrating the increase in IOP with increase in IAH (Lentschener et al., 1996; Mowafi et al., 2003). In the human study (Grosso et al., 2013), induction of standard pressure pneumoperitoneum (IAP, 12–14 mmHg) led to a mild increase in IOP (mean IOP, 4.1 ± 2.9 mmHg). Although the effects of IAH on IOP are still not completely understood, increased IAP has a significant effect on IOP in dogs under conscious conditions without other interventions.

A previous study demonstrated that an increase in IAP to 25 mmHg markedly reduced plasma exogenous creatinine clearance in anaesthetized dogs but had no significant effect in conscious dogs. As described above, general anaesthesia may affect the results of IAH experiments. Although the effect of general anaesthesia on IOP is not clinically significant, propofol has been reported to cause a transient increase in IOP in comparison with baseline values, while IOP remained within normal limits in healthy dogs anaesthetized with sevoflurane (Almeida et al., 2004, 2008; Hasiuk et al., 2014; Kovalcuka et al., 2013). In the present study, an acute increase in IAP to 15 and 25 mmHg in conscious dogs caused a mild increase in IOP, corresponding to a 32.4% and 31.7% increase from baseline values, respectively.

One interesting finding of the present study was that no significant difference was observed in IOP between IAP levels of 15 and 25 mmHg, which indicates that an increase in IAP beyond the initial level did not lead to a corresponding increase in IOP. This result might be attributable to autoregulation of IOP in response to increases in IAP. Nevertheless, it is not clear whether the constancy of IOP despite further increase in IAP is related to the effect of IAH, autoregulation of IOP or both. Although there is no data regarding the effect of IAH on canine IOP, these results suggest that increase in IAP beyond a certain level does not have a clinically significant effect on IOP in healthy dogs. Further studies are required to establish the effect of IAH on IOP.

Decompression of the abdomen commonly results in a decrease in systemic vascular resistance (Grosso et al., 2013; Hunter & Damani, 2004). In the present study, increase in IAP to 15 and 25 mmHg resulted in a significant increase in IOP, which decreased and returned to the baseline level after decompression of the abdomen.

This study has some limitations. Although IAH can affect other body systems, the cardiovascular and respiratory parameters were not evaluated in the present study. A previous study demonstrated that an acute increase in IAP induced discomfort, vomiting, and increased respiratory effort, but cardiovascular parameters and partial pressures of oxygen and carbon dioxide did not show significant differences in conscious dogs (Jang et al., 2018). Although there are only a few studies associating blood pressure with IOP in dogs, existing research demonstrates that moderate alterations in blood pressure have little effect on IOP (Almeida et al., 2004; Macri, 1961). Another limitation is that central venous pressure was not measured in this study. In this study, increased IAP of 15 and 25 mmHg caused a significant increase in IOP in dogs. A possible explanation for this result is that increased IAP might lead to decreased cerebral venous outflow because of increased intrathoracic and central venous pressure, thus causing an increase in IOP (Hunter & Damani, 2004). Although the absence of central venous pressure is definitely a limitation, there is little information about the effect of IAH on IOP in dogs without other interventions and this study provides useful information regarding the effects of IAH on IOP.

## 5 CONCLUSIONS

The present findings demonstrate that increased IAP has a significant effect on IOP in conscious dogs. Because this study was minimally influenced by general anaesthesia, surgical procedures and pneumoperitoneum, these findings enhance our understanding of the effect of IAH on IOP under conscious conditions in dogs. Although more research is needed to determine the effects of increased IAP on IOP, these findings suggest that an acutely increased IAP leads to mild and reversible increases in IOP.

## CONFLICTS OF INTEREST

The authors report no conflicts of interest.

## AUTHORS’ CONTRIBUTIONS

Data collection and writing of the paper were done by Min Jang; Inhyung Lee performed conception and design; Won‐gyun Son, Hyunseok Kim, and Chi Won Shin performed analysis and interpretation; critical revision and final approval of paper were done by Inhyung Lee.

## AUTHOR CONTRIBUTION

Min Jang: Conceptualization; Data curation; Formal analysis; Investigation; Methodology; Project administration; Validation; Visualization; Writing‐original draft; Writing‐review & editing. Won‐gyun Son: Investigation; Methodology; Validation; Writing‐review & editing. Hyunseok Kim: Data curation; Investigation; Methodology; Writing‐review & editing. Chi Won Shin: Data curation; Investigation; Methodology; Validation; Writing‐review & editing. Inhyung Lee: Conceptualization; Funding acquisition; Investigation; Project administration; Resources; Supervision; Writing‐review & editing.

## ETHICAL STATEMENT

The authors confirm that the ethical policies of the journal, as noted on the journal's author guidelines page, have been adhered to and the appropriate Ethical Review Committee approval has been re‐ceived. The Korean National Research Council's guidelines for the Care and Use of Laboratory Animals were followed.

### Peer Review

The peer review history for this article is available at https://publons.com/publon/10.1002/vms3.441.
